# Insights on *Klebsiella pneumoniae* Biofilms Assembled on Different Surfaces Using Phenotypic and Genotypic Approaches

**DOI:** 10.3390/microorganisms5020016

**Published:** 2017-04-03

**Authors:** Maria Bandeira, Vítor Borges, João P. Gomes, Aida Duarte, Luisa Jordao

**Affiliations:** 1 Instituto Nacional de Saúde Dr Ricardo Jorge, Departamento de Saúde Ambiental, Unidade de Investigação e Desenvolvimento-Lisboa, Avenida Padre Cruz, 1649-016 Lisboa, Portugal; mariamfbandeira@gmail.com; 2 Universidade de Lisboa, Instituto Superior Técnico, Departamento de Engenharia Química, Avenida Rovisco Pais, 1049-001 Lisboa, Portugal; 3 Instituto Nacional de Saúde Dr Ricardo Jorge, Departamento de Doenças Infeciosas, Núcleo de Bioinformática, Avenida Padre Cruz, 1649-016 Lisboa, Portugal; vitor.borges@insa.min-saude.pt (V.B.); j.paulo.gomes@insa.min-saude.pt (J.P.G.); 4 Universidade de Lisboa, Faculdade de Farmácia, Av Prof Gama Pinto, 1649-003 Lisboa, Portugal; aduarte@ff.ul.pt

**Keywords:** biofilms, *Klebsiella pneumoniae*, electron microscopy, extracellular polymeric substances (EPS), whole genome sequencing (WGS), healthcare associated infections (HAIs)

## Abstract

*Klebsiella pneumoniae* is a prominent etiological agent of healthcare associated infections (HAIs). In this context, multidrug-resistant and biofilm-producing bacteria are of special public health concern due to the difficulties associated with treatment of human infections and eradication from hospital environments. Here, in order to study the impact of medical devices-associated materials on the biofilm dynamics, we performed biofilm phenotypic analyses through a classic and a new scanning electron microscopy (SEM) technique for three multidrug-resistant *K. pneumoniae* isolates growing on polystyrene and silicone. We also applied whole-genome sequencing (WGS) to search for genetic clues underlying biofilm phenotypic differences. We found major differences in the extracellular polymeric substances (EPS) content among the three strains, which were further corroborated by in-depth EPS composition analysis. WGS analysis revealed a high nucleotide similarity within the core-genome, but relevant differences in the accessory genome that may account for the detected biofilm phenotypic dissimilarities, such as genes already associated with biofilm formation in other pathogenic bacteria (e.g., genes coding haemogglutinins and haemolysins). These data reinforce that the research efforts to defeat bacterial biofilms should take into account that their dynamics may be contingent on the medical devices-associated materials.

## 1. Introduction

In the last decade, multidrug-resistant *K. pneumoniae* isolates have emerged, including strains with additional resistance to carbapenems and colistin, the last line of antibiotic therapy against Gram-negative infections, positioning *K. pneumoniae* as a worldwide public health threat [1,2,3]. In Portugal, this is a major concern, as the isolation of multi-drug resistant *K. pneumoniae* strains has been steadily increasing since 2010 [4]. Pathogenicity factors such as capsules, lipopolysaccharides and fimbriae are important for virulence of *Klebsiella pneumoniae* subsp. *pneumoniae* in both community and healthcare-associated infections (HAI). HAI are the most frequent adverse event in health-care delivery worldwide affecting hundreds of millions of patients per year, leading to significant mortality and financial loses [3].

Biofilm formation is a successful survival strategy for microorganisms known to play a central role in HAIs, especially in medical device associated infections [5]. Bacteria organized in biofilms are difficult to eradicate with common decontamination practices, are generally antimicrobial drugs resistant and could modulate the host immune system [6]. Virulence factors associated with biofilm formation, such as type 1 and type 3 fimbriae, have been implicated in biofilm assembly [7,8,9]. These fimbriae have been identified in *K. pneumoniae* isolates from the gastrointestinal tract and are associated with pneumonia and urinary tract infection. On the other hand, biofilm formation can also be attributed to the fact that *K. pneumoniae* possesses an extremely thick, hypermucoviscous, extracellular polysaccharide capsule. The capsule, a universal feature of clinical and environmental *K. pneumoniae* isolates, is one of major virulence determinants protecting the bacteria from phagocytosis and destruction by antimicrobial peptides [2,10,11].

In order to prevent formation and eradicate existent biofilms, it is crucial to understand the molecular mechanisms involved in biofilm homeostasis, and also how biofilms are affected by their substrate, such as medical devices. In this study, we selected three multidrug resistant *K. pneumoniae* strains identified as etiological agents of HAI, which harbour distinct capsular and fimbrial virulence factors and showed ability to assemble biofilms. The main objective was to compare the *K. pneumoniae* biofilms assembled on the model surface polystyrene cell culture plate and on silicone. Whole-genome sequencing (WGS) was also carried out to search for genetic differences potentially related to biofilm phenotypic differences.

## 2. Materials and Methods

### 2.1. Bacterial Strains

Three *K. pneumoniae* clinical isolates, Kp45, Kp703, Kp2948, from inpatients with HAIs, were identified from skin, urine from indwelling catheter and surgical wound, respectively. These isolates were conserved at −80 °C since 1980 (Kp45 and Kp703) and 2010 (Kp2948) and are part of bacteria collection of Faculty of Pharmacy ULisboa, Portugal.

### 2.2. Biofilms

#### 2.2.1. Biofilm Assembly on Silicone

The assay was performed in triplicate using 6-well flat-bottomed cell culture plates (Nunc, New York, NY, USA) as described previously with small modifications [9]. Briefly, *K. pneumoniae* suspensions at a final concentration of 10^7^ CFU/mL were prepared in 0.9% sodium chloride from fresh cultures in Mueller-Hinton (MH) agar and tenfold diluted in MH broth (Oxoid, Basingstoke, UK). Three millilitres were distributed per well containing silicone disks with 0.75 mm diameter and 20 mm thickness (Sigma, St. Louis, MO, USA). MH broth was used as a negative control. The plates were incubated at 37 °C to allow biofilm formation for different time periods.

#### 2.2.2. Effect of Enzymatic Treatment on Biofilm Formation

Biofilms of *K. pneumoniae* prepared as described in Section 2.2.1 using a volume of 200 μL were grown over night (~20 h) on 96-well flat-bottomed (Nunc) cell culture plates (polystyrene). Wells were vigorously washed three times with sterile distilled water to remove non-adherent bacteria, filled with 100 μg/mL DNaseI (5-Prime/Termofisher, Hilden, Germany) in PBS, 100 μg/mL proteinase K (Applichem, Darmstadt, Germany) or 1.2 units β-*N*-acetylglucosaminidase (Sigma, St. Louis, MO, USA) in acetate buffer pH 5.0 and further incubated at 37 °C for 1 h (DNaseI and proteinase K) or 2 h (β-*N*-acetylglucosaminidase) as previously described [12,13]. Biofilms incubated with PBS or acetate buffer pH 5.0 were used as controls. After incubation wells were washed once with sterile distilled water and stained for 15 min with 100 μL 1.4% crystal violet at room temperature, washed with distilled water three times to remove excess dye and allowed to dry at room temperature. The crystal violet was dissolved in 100 μL of 95% ethanol (Merck, Darmstadt, Germany) and the optical density at 570 nm was read using a SpectraMax 340PC (Molecular Devices, Sunnyvale, CA, USA).

#### 2.2.3. Congo Red-Base Colony Morphology and Transmission Electron Microscopy (TEM)

One microlitre loop of 20 h old biofilms grown on silicone, polystyrene (cell culture dishes) and *K. pneumoniae* cultures in MH broth were spotted onto LB agar plate containing 25 mg/mL Congo red (Sigma). Cultures were incubated for 72 h at 37 °C for colony morphology analysis as described before [14]. Colonies were further analysed by transmission electron microscopy (TEM) under a Morgagni 268D TEM (FEI Electron Optics, Hillsboro, OR, USA) after negative staining with 0.5% uranyl acetate (EMS, Hatfield, PA, USA) in water for 5 min at room temperature. Digital images were acquired with a CCD Mega-View camera (Olympus Soft Imaging Solutions GmbH, Munich, Germany).

#### 2.2.4. Scanning Laser Confocal Microscopy

Biofilms grown on glass coverslips in 24 wells cell culture plates (Nunc) in the conditions described in Section 2.2.1 using a volume of 1 mL per well were stained with calcofluor (Sigma) following the manufacturer instructions. After washing the coverslips were mounted on glass slides and analysed under a SP2 confocal microscope (Leica, Wetzlar, Germany).

#### 2.2.5. Scanning Electron Microscopy (SEM)

Biofilms assembled on polystyrene and silicone disks for 4, 12 and 24 h were processed as previously described for SEM in topographic mode [9].

Biofilms assembled on silicone disks were also prepared for SEM on backscattered electron mode as described before [9] but instead of being sectioned with a diamond knife were prepared as metallographic samples, by grinding and polishing. Grinding was performed using a 400, 600, 1800 and 1200 grit SiC paper (Pace technologies, Tucson, AZ, USA). Polishing was performed with 6-, 3- and 1-micron diameter diamond particles (Microdiamand AG, Lengwil, Switzerland). Both grinding and polishing were performed on a polisher at 150 rpm. Samples were cleaned with 70% ethanol and dried with hot air, mounted on the sample holder with carbon tape, sputter-coated with carbon (20 nm) using a Sputter Coater QISOT ES (Quorum Technologies, Laughton, UK) and analyzed under an electron microscope, JSM-7100F (JEOL, Tokyo, Japan). Micrographs of nonconsecutive areas of biofilms obtained in independent experiments were used to calculate biofilm, bacteria and EPS areas using Image J software.

### 2.3. Statistical Analysis

The results of at least three independent experiments were expressed as the means ± standard deviation (SD). SEM micrographs were analysed using Image J software with the statistical significance assessed by the Student *t*-test (two-tailed). A *p*-value of <0.05 (*) and <0.01 (**) were considered statistically significant.

### 2.4. Whole-Genome Sequencing and Comparative Analyses

Genomic DNA was extracted from pure cultures of the three *K. pneumoniae* isolates (Kp703, Kp45 and Kp2948) using a silica-based automatic DNA extractor (EasyMag). The yield and quality of the purified DNA were assessed with a Qubit assay (Quant-iT double-stranded DNA [dsDNA] assay kit, broad range; Life Technologies, MA, USA) and agarose gel (0.7%) electrophoresis. Nextera XT Illumina libraries were then prepared from high-quality DNA samples and subsequently subjected to paired-end sequencing on a MiSeq equipment (Illumina Inc., San Diego, CA, USA), according to the manufacturer’s instructions. FastQC version 0.11.3 (Babraham Institute, Cambridge, UK) (http://www.bioinformatics.babraham.ac.uk/projects/fastqc/) and FASTX version 0.0.13 (Cold Spring Harbor Laboratory, New York, NY, USA) (http://hannonlab.cshl.edu/fastx_toolkit/) software tools were applied to evaluate and improve the quality of the raw read sequence data, respectively. Subsequently, draft *K. pneumoniae* genomes were de novo assembled using Velvet version 1.2.10 (EMBL-EBI, Hinxton, UK) (https://www.ebi.ac.uk/~zerbino/velvet/) by taking advantage of the VelvetOptimiser script version 2.2.5. Obtained draft genome sizes were 5.5 Mbp for Kp703 and Kp45 isolates, and 5.7 Mbp for the Kp2948 isolate. A preliminary pan-genome overview on the coding sequence (CDS) homology and content was deduced using RAST version 2.0 (Fellowship for Interpretation of Genomes, Burr Ridge, IL, USA) annotation (http://rast.nmpdr.org/) and subsequent sequence-based comparison, where proteins with sequence identity above 75% were considered as homologues. Subsequently, a detailed presence/absence analysis of the accessory genome was achieved through both the identification of positional homologous CDSs and carefully inspection of large insertions and deletions (indels) in whole-genome alignments constructed using the progressive algorithm of Mauve software version 2.3.1 (University of Technology Sydney, Sidney, Australia) (http://darlinglab.org/mauve/mauve.html). For confirmation purposes, reads were mapped using Bowtie2 version 2.1.0 (Johns Hopkins University, Baltimore, MD, USA) (http://bowtie-bio.sourceforge.net/bowtie2/index.shtml) against all detected genomic regions of the accessory genome. Finally, the SNP density within the core-genome was also evaluated through the extraction of extended MAUVE core-alignments (encompassing both coding and non-coding regions) by keeping and concatenating regions where whole-genome sequences aligned over at least 500 bp. Overall nucleotide distances in these extended core-genome alignments were then calculated using MEGA5 (http://www.megasoftware.net). Other in silico analyses performed over the draft genome sequences included: (i) MLST using the ST extraction tool available at the Bacterial Isolate Genome Sequence Database (BIGSdb) (Institut Pasteur, Paris, France) (http://bigsdb.web.pasteur.fr/); (ii) prediction of putative antimicrobial resistance (AMR) genes using both CARD (https://card.mcmaster.ca/) and ResFinder (Center for Genomic Epidemiology, DTU, Kemitorvet, Denmark) (http://www.genomicepidemiology.org/); and (iii) prophage prediction using PHAST (University of Alberta, Edmonton, Canada) (http://phast.wishartlab.com/).

### 2.5. Nucleotide Sequence Accession Numbers

Raw sequence reads of each *K. pneumoniae* isolate were deposited in Sequence Read Archive (SRA) under the accession numbers: SRR4046826 (for Kp703), SRR4046827 (for Kp45) and SRR4046828 (for Kp2948). Draft genome sequences were also submitted to DDBJ/ENA/GenBank, and are available under the accession numbers: MDUJ00000000 (for Kp703), MDUK00000000 (for Kp45) and MDUL00000000 (for Kp2948).

## 3. Results

### 3.1. Biofilm Assembly on Polystyrene and Silicone

**Figure 1:**
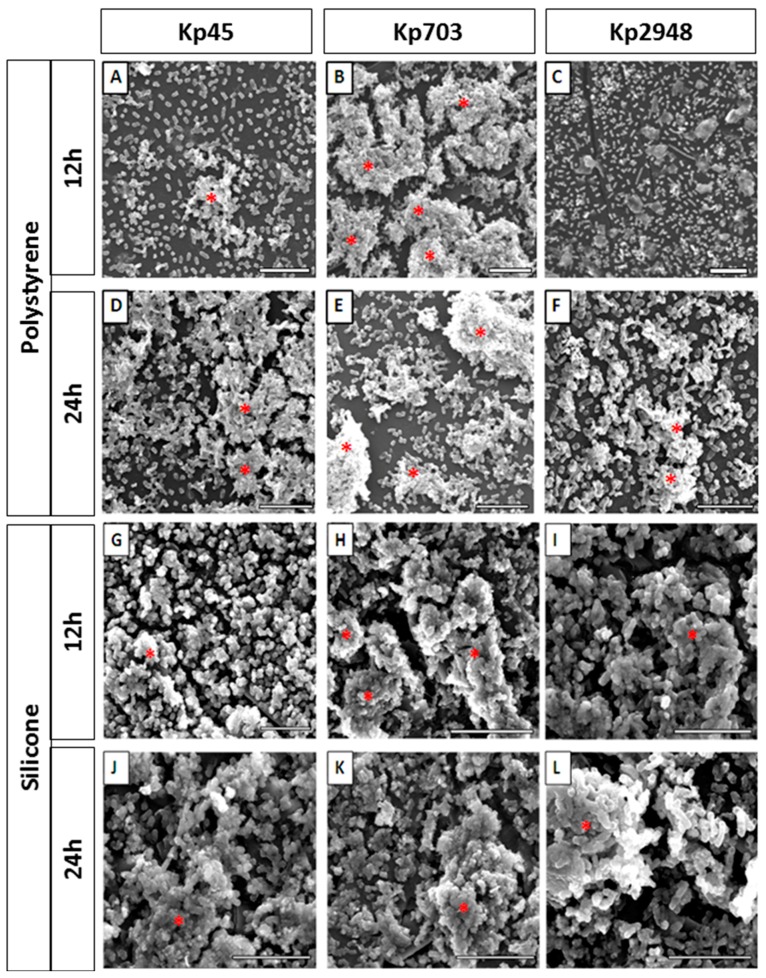
*K. pneumoniae* biofilms assembled on different surfaces: (**A**–**F**) representative micrographs of 12 h and 24 h old biofilms assembled on polystyrene; and (**G**–**L**) silicone by Kp45, Kp703 and Kp2948 are shown. Twelve hours old polystyrene biofilms: (**A**) Kp45; (**B**) Kp703; and (**C**) Kp2948; 24 h old polystyrene biofilms: (**D**) Kp45; (**E**) Kp703; and (**F**) Kp2948; 12 h old silicone biofilms: (**G**) Kp45; (**H**) Kp703; and (**I**) Kp2948; and 24 h old silicone biofilms: (**J**) Kp45; (**K**) Kp703; and (**L**) Kp2948. The structures identified as mature biofilms are highlighted with a red asterisk (*). Scale bars = 10 µm.

The biofilm assembly of three *K. pneumoniae* clinical isolates, Kp45, Kp703 and Kp2948, on polystyrene surfaces were determined previously. The results showed the best Kp703, the worse Kp2948 biofilm assemblers and the Kp45 isolate was at intermediate position [9]. In this study, in addition to polystyrene (Figure 1A–F) biofilm assembly was also monitored on silicone (Figure 1G–L) using scanning electron microscopy (SEM) in topographic mode. On polystyrene surfaces, after 12 h, structures compatible with mature biofilms were identified for Kp45 (Figure 1A) and Kp703 (Figure 1B) but not for Kp 2948 (Figure 1C). Micrograph analysis clearly shows that Kp703 is the best biofilm assembler exhibiting well organized and denser biofilms. After 24 h an increase on biofilm formation was observed for Kp45 (Figure 1D) and Kp2948 (Figure 1F) whereas for Kp703 (Figure 1E) the opposite was observed. On silicone surfaces, biofilms were observed for all bacteria after 12 h (Figure 1G–I) and an increase in biofilm density was observed at 24 h (Figure 1J–L). Despite this difference, Kp703 was the best biofilm assembler on both surfaces.

**Figure 2:**
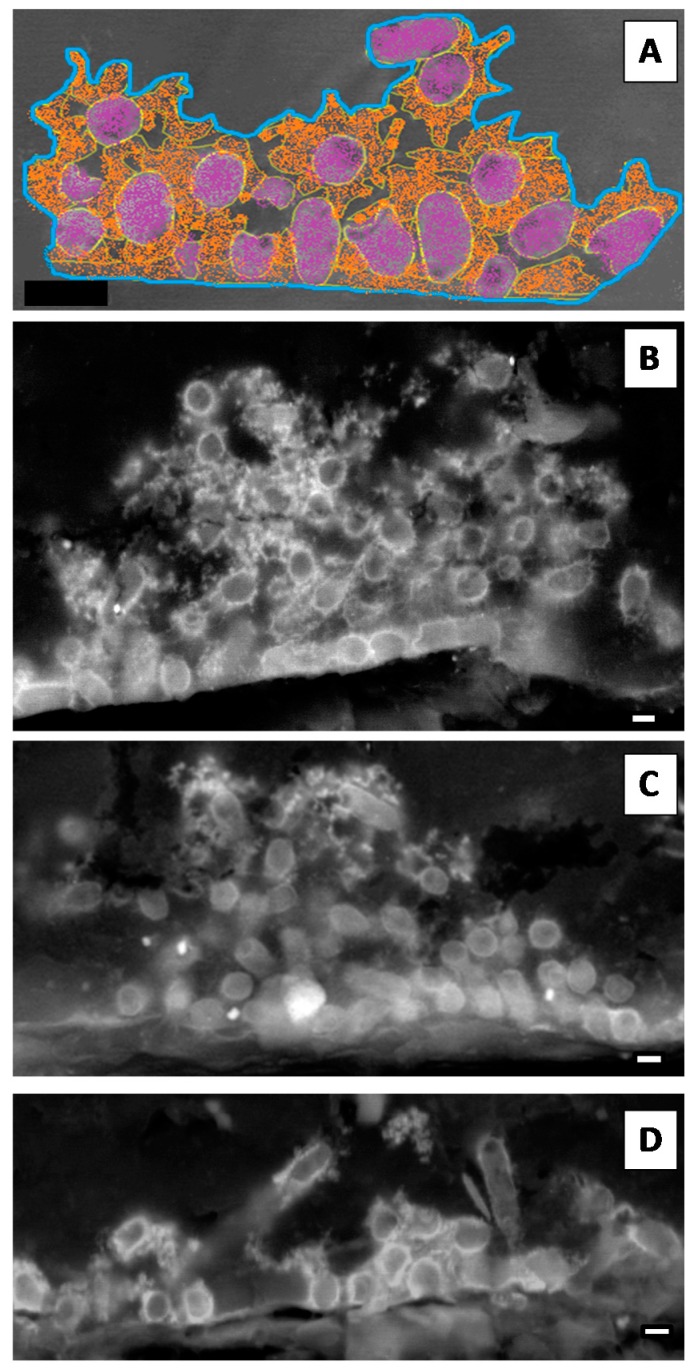
*K. pneumoniae* biofilms assembled on silicone. The relative areas occupied within biofilms by its major components (bacteria and extracellular polymeric matrix—EPS) were determined using ImageJ software. (**A**) The total biofilm area was delimited (blue line) as well as the areas occupied by bacteria (purple) and EPS (orange) as schematically shown. Representative micrographs of 4 h old: (**B**) Kp703; (**C**) Kp45; and (**D**) Kp2948 are shown (scale bars = 1 µm).

**Figure 3:**
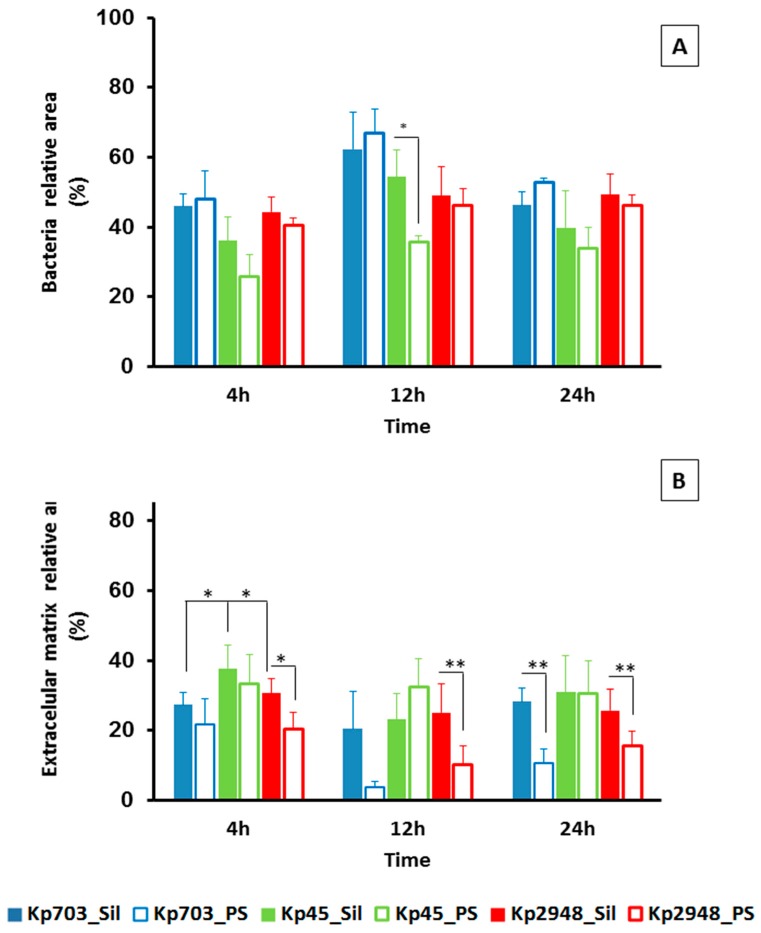
Characterization of *K. pneumoniae* biofilms assembled on silicone. (**A**) Bacteria; and (**B**) EPS relative areas existent on 4 h, 12 h and 24 h old biofilms assembled on silicone by the three isolates were determined. Bold bars were used for biofilms assembled on silicone and empty bars for biofilms assembled on polystyrene (results from [9]). (**A**) A significant statistical difference in bacteria relative area was observed for 12 h old for Kp45 biofilms assembled on polystyrene and silicone. (**B**) For extracellular matrix relative areas, significant statistical differences were observed for 4 h, 12 h and 24 h old Kp2948 assembled on the two surfaces and also between 4 h old Kp45 biofilms assembled on silicone and the other two isolates (* *p* < 0.05; ** *p* < 0.01).

In terms of their major components, we compared biofilms assembled on polystyrene and silicone: biomass (bacteria) and extracellular polymeric matrix (EPS) were calculated according to the relative areas occupied by each component. Biofilms with different ages were cross-sectioned and analysed by SEM in backscattered electron mode. Although for biofilms assembled on polystyrene this was a straightforward procedure, as previously described [9], for biofilms assembled on silicone a new procedure had to be established based on material sciences samples preparation protocols. This strategy allowed us to cross-section the biofilm and to determine the relative areas occupied by bacteria and EPS using ImageJ software (Figure 2A). Representative micrographs of 4 h old biofilms of Kp703 (Figure 2B), Kp45 (Figure 2C) and Kp2948 (Figure 2D) corresponding to the adherence phase are shown and at this early stage, the biofilms assembled by the three bacteria are different. The results obtained for the relative areas occupied by biomass (Figure 3A) and EPS (Figure 3B) within biofilms assembled on silicone are showed in Figure 3. For easier comparison, the results previously published for polystyrene were also included although excluding the statistical analysis [9]. No significant differences were found at 4 h, 12 h and 24 h between the bacteria relative areas within biofilms assembled on silicone by the three *K. pneumoniae* isolates (Figure 3A). Nevertheless, the relative areas occupied by bacteria in 12 h old biofilms assembled on silicone by Kp45 were significantly higher than on polystyrene (*p* = 0.042) (Figure 3A). For both Kp703 and Kp2948 no statistically significant differences were found.

Concerning the extracellular matrix relative area, significant differences were found at 4 h (attachment phase) for biofilms assembled on silicone by Kp45 and the other two bacteria (*p* < 0.053) (Figure 3B). In general, larger relative areas were occupied by EPS in biofilms assembled on silicone than on polystyrene for all bacteria within the studied time. For Kp2948, the relative areas occupied by excreted matrix at 4 h (*p* = 0.042), 12 h (*p* = 0.006) and 24 h (*p* = 0.013) within biofilms were statistically different between polystyrene and silicone (Figure 3B).

### 3.2. The Extracellular Polymeric Matrix (EPS)

**Figure 4:**
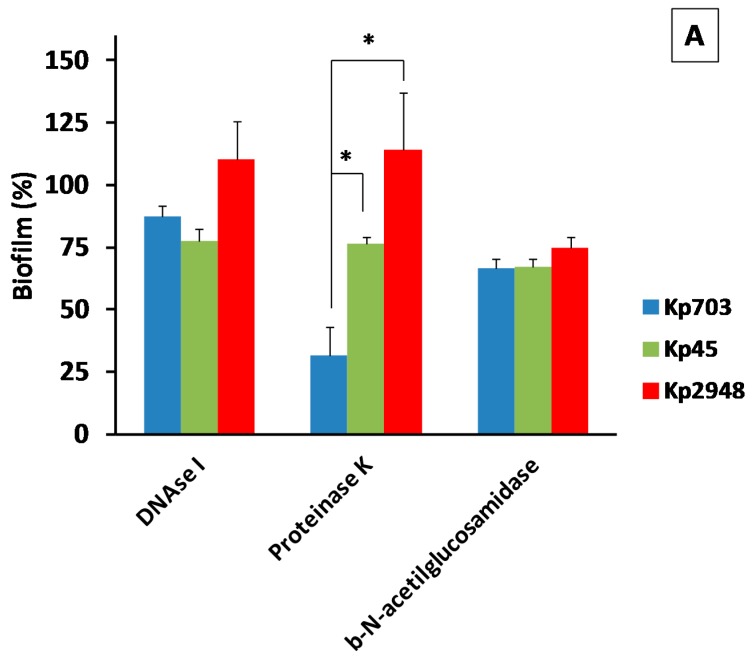

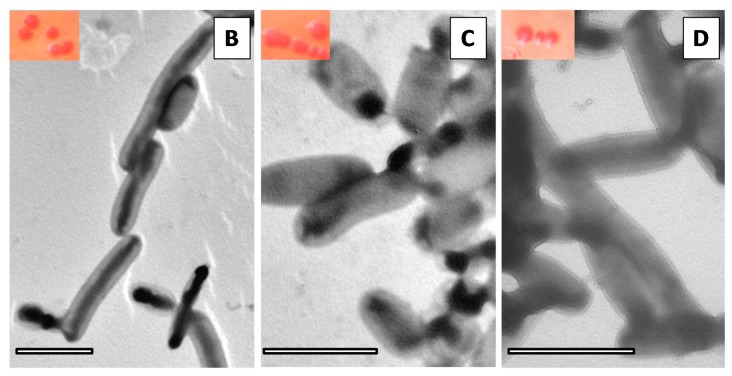
The extracellular polymeric matrix of *K. pneumoniae* biofilm. (**A**) The results of enzymatic digestion of Kp biofilms are presented (* *p* < 0.05). In the insets of figures (**B**–**D**) are shown CFU of: (**B**) Kp703; (**C**) Kp45; and (**D**) Kp2948 grown on MH-Congo red after recovery from biofilms assembled on silicone. In the TEM micrographs of: (**B**) Kp703; (**C**) Kp45; and (**D**) Kp2948, it is possible to observe the absence of curli (scale bars = 2 µm).

Enzymatic dispersion of biofilms is one of the methods used to elucidate the nature of the EPS produced by a specific microorganism. The enzymes DNase I, proteinase K and β-*N*-acetylglucosaminidase were used to disperse mature biofilms assembled on polystyrene instead of silicone because the EPS structure for all *K. pneumoniae* strains on this surface were less prominent than on polystyrene. The biofilm dispersion by β-*N*-acetilglucosamidase is more effective for all isolates (Figure 4A) than by DNase I suggesting that the EPS could be richer in exopolysacharides than in secreted nucleic acids. The major difference in biofilm dispersion was observed for proteinase K. A significant difference in biofilm digestion by this protease was observed between Kp703 and the other isolates (*p* < 0.044). In addition, the Kp703 isolate displayed a significant difference in biofilm digestion by proteinase K when compared with the other two enzymes (*p* = 0.020), indicating that EPS protein content should be particularly high around 65%. Another curious observation is the similar digestion rate detected for all enzymes on Kp45 biofilm which exhibited higher relative areas of EPS.

The lack of staining by calcofluor of mature *K. pneumoniae* biofilms showed that cellulose is either not present or is a minor EPS component. Since calcofluor binds specifically to cellulose but not to curli fimbriae, congo red was used to investigate the presence of curli in *K. pneumoniae* recovered from biofilms assembled both on polystyrene and silicone. Independently of the *K. pneumoniae* strain at 72 h the colonies are mucoid with only a slight difference in colour. The reddish colour of Kp703 and Kp45 is similar and darker than that of Kp2948 for both substrates. Representative images of Kp703, Kp45 and Kp2948 isolates are shown in the insets at Figure 4B–D, respectively. To look for curli, bacterial colonies were further analysed by TEM after negative staining. Curli were absent, as shown for Kp703 (Figure 4B), Kp45 (Figure 4C) and Kp2948 (Figure 4D).

### 3.3. Whole-Genome Analysis of K. pneumoniae Isolates Displaying Different Biofilm Phenotypes

**Figure 5:**
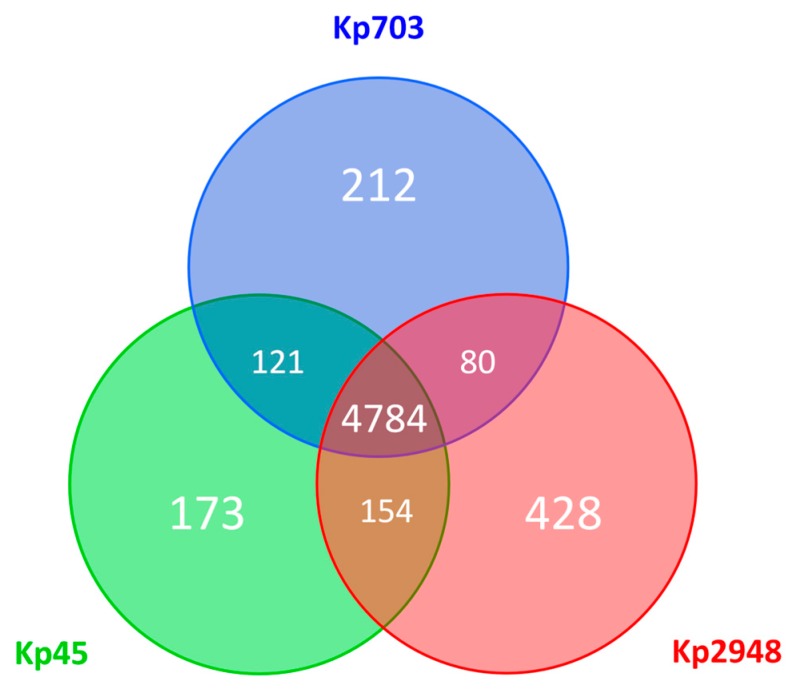
Pan-genome overview. The Venn diagram shows the number of shared and unique predicted coding sequences (CDSs) among the three *K. pneumoniae* isolates (Kp703, Kp45 and Kp2948) deduced from a Basic Local Alignment Search Tool (BLASTP) analysis performed using RAST, where protein sequences with more than 75% of sequence identity were considered as homologues. Circles are not drawn to scale.

The analysis of the genetic diversity within the core-genome (encompassing about 90–94% of each whole-genome sequence) revealed a nucleotide similarity above 99.5% (20172/5151664 variant sites). Still, relevant genetic differences (detailed in Table 1 and Supplementary Table S1) were found when assessing the accessory genome of the three *K. pneumoniae* strains (a general overview on the pan-genome is provided in Figure 5). While the Kp45 strain-specific accessory genome is overrepresented by prophage-like elements, the accessory genome of the isolate collected 30 years later (Kp2948) is marked by both prophage-like elements and putative antimicrobial resistance (AMR) genes encoded both in the chromosome and plasmids (Table 1).

A major concern related to *K. pneumoniae* is the association of hypervirulence with multi-drug resistance to generate high-risk strains [15]. Multilocus sequence typing (MLST), based on 7 housekeeping genes, has been used to recognize *K. pneumoniae* clonal groups and identify their medically relevant features [16]. Kp703 belongs to ST15, whereas both Kp45 and Kp2948 belong to ST14. The ST14 differs from ST15 by only 1 allelic mismatch (in *infB*), so both STs are included in same clonal complex (CC) CC14. Similar to what has been observed in previous large-scale genome analyses of the diversity and population structure of *K. pneumoniae* [17], we have also found a huge genome-based relatedness between ST14 and ST15 isolates. Several genetic loci have been identified as determinants of adhesion and virulence in all three *K. pneumoniae* clinical isolates.

## 4. Discussion

The ability of three *K. pneumoniae* responsible for HAIs, identified with a time interval of 30 years, to assemble biofilms on different surfaces and the interplay between biofilm phenotype and bacterial genome were the focus of the present study. The characteristics of these *K. pneumoniae* multi-drug resistant isolates producing β-lactamases were described by Bandeira and colleagues [9]. Briefly, all three strains produce an AmpC-type class C and SHV-28 class A β-lactamases, in addition Kp2948 isolate produces the KPC-3 carbapenemase encoded in a plasmid. The progressive acquisition of AMR genes could be explained by the abundant antibiotic exposure occurred during the last decades, and plausibly explains the extended accessory genome observed for Kp2948 when comparing with the two 1980s isolates. Kp45 identified in 1980 and Kp2948 in 2010 had the capsular serotype K2 while Kp703 strain, also identified at 1980, was non-capsulated and the O antigen was type O:1. Bacterial genome analysis showed that the cps region of serotype K2 capsular polysaccharide synthesis was different of Kp703 non-capsulated. The DNA sequences of four open reading frames (*wzi*, *wza*, *wzb*, and *wzc*) from *cps* loci responsible by capsular antigen translocation and cell surface assembly were compared. The *wzb* gene was not found in Kp703 strain. The absence of *wzb* in this strain could explain the non-capsular characteristic and is consistent with the work of Pan and colleagues [18].

The three strains can be placed according to biofilm assembly on polystyrene and on silicone in ascending order as follows: Kp2948, Kp45 and Kp703. Biofilm assembly is a complex process that is not only dependent on the bacterial strain but also on the nature of the surface where it is assembled and the conditioning media [19]. All these factors influence not only biofilm formation but also the nature of the biofilm assembled. In the present study, biofilms assembled on silicone were denser than those assembled on cell culture plate. This difference in biofilm density is dependent on both bacteria and biofilm assembly stage. Nevertheless, silicone biofilms disperse at a slower rate than polystyrene biofilms being this feature particularly evident for Kp703, which was identified from a burn patient with a catheter-associated urinary tract infection. A slower dispersion rate favours bacterial persistence on catheters which can function as reservoirs of bacteria, with special relevance for urinary tract infections, the main cause of HAIs. It has already been shown that bacteria able to assemble biofilm are more often responsible for chronic infections [20].

The comparison between biofilms assembled on polystyrene and silicone showed that the three bacteria behave differently. As described in the literature bacterial biofilms can display many phenotypes, being influenced by surface features and microorganisms structure [21]. The ability of Kp703 to assemble biofilm was not affected by the surface nature (polystyrene/silicone). On the other hand, the Kp2948, less prone to assemble biofilms, increased its performance on silicone. The same behaviour was observed previously for these strains on metal [22].

Differential EPS secretion on polystyrene and silicone might play a crucial role in the development of different biofilm phenotypes. As reported previously EPS influences biofilm assembly and is particularly important to attachment and dispersion phases [23,24,25]. Although the relative areas occupied by EPS for all bacteria were higher on silicone than on polystyrene, the difference was statistically significant only for Kp2948 (*p* < 0.05). This could account for the existence of Kp2948 biofilms with different organizations in both materials. Different compounds have been identified at EPS composition such as proteins, humic substances, metal ions, exopolysacharides and nucleic acids, as parts of the cement that holds together bacteria within the well-defined structure of biofilm [26,27]. Our data suggests that for *Klebsiella* strains the principal differences at composition of EPS are on protein content.

Curli fimbriae and cellulose have been identified as major EPS components among *Enterobacteriaceae* facilitating cell adhesion during symbiotic or infectious interactions and for bacterial adherence to abiotic surfaces [28]. Nevertheless, even though *K. pneumoniae* is an enterobacterium, our data showed that these components were not detected within biofilms of the three isolates. Genes required for curli fimbriae expression are clustered in *csgBA* operon that encodes the structural components, whereas the divergently oriented *csgDEFG* operon encodes proteins involved in curli assembly and transport. Curli fimbriae biosynthesis is commonly regulated by *csgD* gene, a transcriptional activator, which is necessary for *csgBA* transcription [29]. Comparative genome analysis of the three strains showed that only the *csgD* gene is missing from the *csgBA* operon which could justify the absence of curli fimbriae. For cellulose biosynthesis, the genes are encoded by the *bcsABC* operon that is found in some *K. pneumoniae* strains and *Raoultella ornithinolytica*, which carry both type I (*bcsABCO*) and type II *bcs* operons [30]. BcsO are proline-rich proteins of unknown function encoded in enterobacteria. Our results showed that cellulose synthase operon type I (*bcsABCO* genes) was carried in all three strains in duplicate, what led us to hypothesize that the phenotypic method lacked accuracy to detect the cellulose.

The attachment of microorganisms is the first step of biofilm assembly. It is a complex process being regulated by diverse factors such as growth medium, substratum, and cell surfaces. As previously mentioned biofilms are associated with device-related infections a type of HAIs of major importance for public health. Urinary tract infections acquired in the hospital are catheter-associated. The presence of an indwelling catheter provides a site for bacterial attachment and facilitates long-term colonization. For all these reason is important to investigate how the genome of our three isolates could influence this process. Attachment to abiotic surfaces and host cell surfaces is mediated by Type 1, Type 3 and Type IV fimbriae. Type 1 fimbriae in *K. pneumoniae* are encoded on a gene cluster (*fim*) containing all the genes required for the fimbrial structure and assembly, and play a crucial role in colonization [31]. The type 3 fimbriae are characterized by their ability to agglutinate erythrocytes treated with tannic acid in vitro, and this phenotype has been referred to as the mannose-resistant *Klebsiella*-like hemagglutination (MR/K) reaction [32]. These fimbriae are encoded by the mrk operon and mediate the initiation of biofilm assembly on surfaces coated with host-derived extracellular matrix proteins and abiotic surfaces. Type IV fimbriae mediate attachment and adherence to epithelial cells, twitching motility, gliding motility, cell agglutination, and biofilm and fruiting body formation. They act as receptors for bacteriophages and they are capable of extension and retraction, processes that are integral to twitching motility [33]. The genes involved in the biosynthesis of surface molecules required for the formation of biofilms, such as capsule, poly-beta-1,6-*N*-acetyl-d-glucosamine (PGA) and fimbria were found in this study (Table 1). The importance of these proteins on biofilm formation was in agreement with previous reports.

For the worst biofilm assembler, Kp2948, we found a significant number of prophage-like elements. This result kept our attention since prophages activation was reported in other microorganism as an inducer of cell death inside microcolonies leading to biofilm dispersion [34]. Nevertheless, a direct link could not be drawn as several unique prophages and prophage-like elements were also found for the best biofilm assemblers Kp703 and Kp45. On the other hand, the accessory genome of the best biofilm assembler Kp703 was found to be enriched by a higher number of signature sequences encoding for molecular players potentially involved in biofilm assembly, as described in other bacterial species. As examples, we highlight the identification of: (i) the gene coding for a filamentous haemoagglutinin, a type of adhesins reported as key factor for *Acinetobacter baumannii* attachment and subsequent cell accumulation on substrates [35]; (ii) a haemolysin expression modulating protein Hha whose homologues in *E. coli* seem to be involved in controlling biofilm formation [36]. The role played by ribose and its transport-system (ribose ABC-transporter system) is not straightforward. Streptococcal biofilm formation was significantly inhibited by ribose and other pentose molecules [37]. In *Aggregatibacter actinomycetemcomitans* oral biofilms, ribose and a quorum sensing molecule (autoinducer 2- AI2) compete to the subunit B of ribose ABC-transporter system [38]. The impact of this interaction on biofilm homeostasis is not fully understood, illustrating the complexity of biofilm assembly.

Regarding other genotype/phenotype associations, both ST14 and ST15 have been associated to multidrug resistance in different countries worldwide [17]. The association with carbapenem resistance is a major problem that has been reported in different wards at Portuguese hospitals [6,39,40], and in Finland [41], the USA [42], and other countries. Although biofilm assembly is known to largely contribute to increased antibiotic resistance, few studies have been performed to relate ST, biofilm and antibiotic resistance. An exception is a study performed by Naparstek and colleagues showing that extremely drug resistant KPC-producing *K. pneumoniae* isolates have relatively low-mass biofilms (OD590 range 0.02–0.3 for 18 h old biofilms) particularly those belonging to the ST258 lineage [43]. A direct comparison cannot be performed, since the lineages ST14 and ST15 were not included and the experimental conditions are slightly different from ours. Nevertheless, we used tenfold diluted inoculum and after 12 h the worst biofilm assembler Kp2948 isolate already had an OD570 of 0.39 that steadily increased until 24 h. It is tempting to argue that these ST14/ST15 lineages are better biofilm assemblers. Future experiments involving other ST isolates will certainly dissect this hypothesis.

Overall, while the well-known genome plasticity of *K. pneumoniae* species hampers the establishment of conclusive and straightforward genotype/phenotype associations, this genomic analysis points some genetic features that may be prioritized for future functional studies, focused on clarifying their involvement in biofilm formation and homeostasis, which provides several clues to design follow up studies of biofilm transcriptome.

## 5. Conclusions

The three *K. pneumoniae* isolates responsible for HAIs could assemble biofilms following the same ranking order on polystyrene (cell culture plate) and silicone. This result simultaneously strength the hypothesis that medical devices work as reservoirs of *K. pneumoniae* for onset infections and validate the use of cell culture lab ware for biofilm studies. Differences in EPS composition were identified between the major biofilm assembler Kp703 and the other two isolates (Kp45 and Kp2948). A genomic analysis of the three *K. pneumoniae* genomes allowed identifying potential players responsible for the biofilm phenotypic differences. These results provided good clues to design further studies namely of biofilm transcriptome and metabolome to fully understand *K. pneumoniae* biofilms.

## Figures and Tables

**Table 1:** Genetic differences in the accessory genome of the *K. pneumoniae* isolates.

Genomic Region ^1^	Approximate Length (Kbp)	Isolate			Relevant Information ^2,3^
		Kp703	Kp45	Kp2948	
**1**	45	**√**	---	---	Intact prophage (predicted by PHAST)
**2**	41	**√**	---	---	Intact prophage (predicted by PHAST)
**3**	4	**√**	---	---	Includes **predicted AMR genes**: tetracycline resistance genes *tet(A/G)* and *tetR*
**4**	6	**√**	---	---	Includes **Ribose ABC-transporter system** proteins (permease RbsC, ATP-binding RbsA)
**5**	11	**√**	---	---	Includes genes encoding a **Filamentous hemagglutinin** and a Hemolysin transporter protein
**6**	57	**√**	---	---	Includes genes encoding a **Haemolysin expression modulating protein**, the YdeA protein and a Programmed cell death antitoxin MazEF
**7**	12	**√**	---	---	Putative **prophage elements**: predicted annotation includes several hypothetical proteins, a purine NTPase and a prophage CP4-57 integrase
**8**	2	**√**	**√**	---	Aerotaxis sensor receptor protein
**9**	13	**√**	**√**	---	Includes an **additional Urea ABC transport system** (another similar gene cluster is carried by all three isolates)
**10 ^4^**	36	**√**	---	---	Incomplete prophage (predicted by PHAST) carrying a **strain-specific Glycosaminoglycan attachment site** encoding gene
**11 ^4^**	36	---	**√**	---	Incomplete prophage (predicted by PHAST) carrying a **strain-specific Transcription activator *gutR* gene**
**12**	12	---	**√**	**√**	Putative plasmid fragments carrying the virulence-associated proteins VagC and VagD
**13**	37	---	**√**	---	Intact prophage (predicted by PHAST)
**14**	43	---	**√**	---	Intact prophage (predicted by PHAST)
**15**	57	**√**	---	**√**	Includes genes encoding **Type IV pili-related proteins**, a TonB-dependent protein, **Iron aquisition yersiniabactin synthesis enzymes**; and a **predicted AMR gene** likely associated with resistance to tetracycline and aminoglycosides (*fyuA*)
**16**	2	**√**	---	**√**	Includes **a predicted AMR gene**: blaTEM-1A—class A beta-lactamase
**17**	13	---	**√**	**√**	Includes genes encoding **Type I secretion related proteins** (LapC, ATPase LapB, LapE, agglutinin RTX)
**18**	7	---	**√**	**√**	Includes **predicted AMR genes**: Aminoglycoside 3′-phosphotransferase strA and strB [or APH(3″)-Ib and APH(6)-Id, respectively] likely associated with resistance to aminoglycosides
**19**	11	---	**√**	---	Predicted annotation includes several hypothetical proteins and putative phage-like elements.
**20**	39	---	**√**	---	Intact prophage (predicted by PHAST)
**21**	17	---	---	**√**	Questionable prophage (predicted by PHAST)
**22**	11	---	---	**√**	Incomplete prophage (predicted by PHAST)
**23**	40	---	---	**√**	Intact prophage (predicted by PHAST)
**24**	25	---	---	**√**	Questionable prophage (predicted by PHAST)
**25**	37	---	---	**√**	Questionable prophage (predicted by PHAST)
**26**	12	---	---	**√**	Incomplete prophage (predicted by PHAST)
**27**	5	---	---	**√**	Includes genes encoding a **putative serine protease** (from the Peptidase S8 Subtilisin superfamily) and a cell division protein FtsH
**28**	27	---	---	**√**	Putative fragment of a conjugative plasmid including genes encoding **Type IV pili-related proteins**, a Programmed cell death antitoxin MazE and a zinc metalloproteinase Mpr protein
**29**	1	---	---	**√**	Includes a **predicted AMR gene**: trimetroprim resistance gene *dfrA14* coding for a Dihydrofolate reductase
**30**	40	---	---	**√**	Intact prophage (predicted by PHAST)
**31**	4	---	---	**√**	Includes **predicted AMR genes**: tetracycline resistance genes *tet(A/G)* and *tetR*; and proteins from the Glutathione-dependent pathway of formaldehyde detoxification
**32**	17	---	---	**√**	Includes a **predicted AMR gene** (KPC-3 beta-lactam resistance gene) and gene encoding a Chromate resistance protein ChrB
**33**	22	---	---	**√**	Includes genes coding for a **arsenic resistance operon** and a outer membrane protein or related peptidoglycan-associated (lipo)protein
**34**	5	---	---	**√**	Includes genes coding for a **phosphonate ABC transport system and the RuBisCO operon transcriptional regulator CbbR**
**35**	1	---	---	**√**	Includes a **predicted AMR gene**: SHV-161 beta-lactam resistance gene
**36**	3	---	---	**√**	Includes a **predicted AMR gene**: AC(6′)-Ib putative fluoroquinolone resistance gene
**37**	2	---	---	**√**	Includes a **predicted AMR gene**: putative Sulphonamide resistance gene *sul1* coding for a Dihydropteroate synthase
**38**	1	---	---	**√**	Includes a **predicted AMR gene**: putative Sulphonamide resistance gene *sul2* coding for a Dihydropteroate synthase

^1^ Locations of each genomic region within the genome contigs are detailed in Supplementary Table S1. **√**, present. ---, absent; ^2^ Annotation features were predicted using RAST (http://rast.nmpdr.org/); ^3^ Putative AMR genes were predicted using both CARD (https://card.mcmaster.ca/) and ResFinder (http://www.genomicepidemiology.org/), while prophage prediction was performed using PHAST (http://phast.wishartlab.com/); ^4^ These two putative prophage elements show considerable homology, but harbour dissimilar strain-specific genes, thus were considered as distinct genomic regions.

## Supplementary Materials

The following are available online at http://www.mdpi.com/2076-2607/5/2/16/s1, Table S1: Detailed location of the genomic regions found in accessory genome of the *K. pneumoniae* isolates.
