# Supplementary material for: Insights on Klebsiella pneumoniae Biofilms Assembled on Different Surfaces Using Phenotypic and Genotypic Approaches
Source: Microorganisms. 2017 Apr 3;5(2):16. doi: 10.3390/microorganisms5020016 (PMC5488087; doi:10.3390/microorganisms5020016)
Supplement: Supplementary file 1 [file microorganisms-05-00016-s001.pdf]

Table S1: Detailed location of the genomic regions found in accessory genome of the *K. pneumoniae* isolates.

Genomic region	Contig nº (bp location) [1]		
	**Kp703**	**Kp45**	**Kp2948**
**1**	14 (33420-78461)	---	---
**2**	17 (31818-73195)	---	---
**3**	19 (1-3882)	---	---
**4**	40 (583642-589716)	---	---
**5**	74 (34702-47865)	---	---
**6**	121 (1-57266)	---	---
**7**	8 (85065-97181)	---	---
**8**	11 (27681-29438)	52 (1-1271)	---
**9**	28 (1-11937); 3 (1-3191)	35 (57896-71598)	---
**10**	56 (191811-227997)	---	---
**11**	---	17 (43644-79326)	---
**12**	---	18 (1-12394)	33 (1-14831)
**13**	---	30 (131058-168299)	---
**14**	---	34 (1-43459)	---
**15**	20 (74425-131652)	---	105 (64308-97514); 227 (1-18761); 191 (1-869); 176 (1-718); 193 (1-2953)
**16**	26 (1-1856)	---	70 (1-1696); 94 (520)
**17**	---	46 (145706-158835)	184 (1-12343)
**18**	---	73 (1-6909)	21 (7549); 217 (1714); 76 (1-375)
**19**	---	108 (18731-29947)	---
**20**	---	108 (144770-183310)	---
**21**	---	---	110 (217714-234854)
**22**	---	---	137 (239588-250991)
**23**	---	---	8 (40463-77928); 137 (367234-368894)
**24**	---	---	150 (1-25438)
**25**	---	---	256 (106917-144254)
**26**	---	---	158 (15631-27237)
**27**	---	---	72 (2392-7596)
**28**	---	---	2 (1-27296)
**29**	---	---	24 (1-1133)
**30**	---	---	195 (35740-64378); 29 (1071); 61 (61399-72132)
**31**	---	---	47 (1-3700)
**32**	---	---	9 (1-16630)
**33**	---	---	79 (1-22765)
**34**	---	---	12 (1-4894)
**35**	---	---	140 (1-846)
**36**	---	---	51 (1-2703)
**37**	---	---	87 (1-1962)
**38**	---	---	78 (1-1442)

[1] Some genomic regions assembled in a single contig for a given isolate were found to be completed but fragmented in several contigs in the genome of other isolate.
